# Malignant Peritoneal Mesothelioma: A Case Report

**DOI:** 10.7759/cureus.42902

**Published:** 2023-08-03

**Authors:** Bárbara Sousa, Joana Silva, Nereida Monteiro, Miguel Romano, Elsa Araújo

**Affiliations:** 1 Department of Internal Medicine, Unidade Local de Saúde do Alto Minho (ULSAM) - Hospital Conde de Bertiandos, Ponte de Lima, PRT; 2 Department of Internal Medicine, Unidade Local de Saúde do Alto Minho (ULSAM), Ponte de Lima, PRT; 3 School of Medicine, University of Minho, Braga, PRT

**Keywords:** diffuse abdominal pain, malignant peritoneal mesothelioma, asbestos, malignancy, mesothelioma

## Abstract

Malignant peritoneal mesothelioma (MPM) is a rare tumor of the serous membranes of the peritoneum and has been linked to exposure to asbestos and other risk factors. The clinical manifestations are vague, with a wide clinical spectrum, predominantly related to the abdominal involvement of the disease. Localized mesothelioma is an uncommon manifestation of the disease. Common symptoms include abdominal pain or abdominal distention, nausea, anorexia, and weight loss. Rarely, patients present with paraneoplastic syndrome. Due to the nonspecific symptoms, many patients already have advanced disease at the time of diagnosis.

The authors report a case of a 75-year-old female patient who presented with symptoms of asthenia, anorexia, progressive paleness, and weight loss lasting five months. She reports later new-onset symptoms of diffuse abdominal pain and diarrhea associated with nausea. Laboratory tests showed anemia, mild leukocytosis, thrombocytosis, elevated C-reactive protein (CRP), and elevated liver enzymes. An abdominal and pelvic computed tomography (CT) scan revealed marked tissue thickening of an irregular and striated configuration of the leaflets and peritoneal reflections in an omental cake pattern, and a chest CT scan showed multiple bilateral pulmonary nodules, suggesting diffuse malignant disease. A CT-guided biopsy of a peritoneal implant was performed, establishing the diagnosis of malignant peritoneal mesothelioma. Due to rapid clinical deterioration, the patient did not receive any systemic treatment, surgery, or radiotherapy and was transitioned to comfort care.

As in the presented case, most cases of MPM have diffuse peritoneal involvement at the time of diagnosis, although extra-abdominal involvement is very rare. This disease presentation is associated with high morbidity and mortality compared to cases of localized disease.

There is no specific imaging diagnostic modality or valuable tumor markers for MPM. Although a CT scan remains important in the diagnostic approach, the changes found are not specific. Radiographically, MPM may present as mesenteric or parietal peritoneal nodules, visceral peritoneal thickening, ascites, or omental masses. Although these features may raise suspicion of MPM, a biopsy is necessary to confirm the diagnosis.

Therefore, due to the rarity of this disease and its nonspecific signs or symptoms, MPM is difficult to diagnose, and the prognosis remains poor.

## Introduction

Malignant peritoneal mesothelioma (MPM) is a rare tumor of the connective tissue, involving the serosal membranes of the peritoneum. It represents the second most common origin of mesothelioma, following the pleura, with 7%-30% of all cases [1].

Mesothelioma is more common in males and has been linked to industrial contaminants and mineral exposure, especially asbestos, although many other risk factors have been included. In some cases, mesothelioma is idiopathic [2-4]. Asbestos remains the main carcinogen associated with pleural mesothelioma; however, in MPM, this association is weaker, with less than 50% of patients reporting previous exposure to asbestos [1].

The main clinical manifestations of MPM are usually related to the abdominal involvement of the disease, with abdominal pain and distension being the main associated complaints [5]. It can also manifest with nonspecific symptoms such as nausea, anorexia, and weight loss.

## Case presentation

The authors report a case of a 75-year-old female patient, with a past medical history of arterial hypertension, dyslipidemia, and diabetes mellitus, with no history of alcohol consumption, smoking, and asbestos exposure. The patient presented with symptoms of asthenia, anorexia, progressive paleness, and weight loss for five months. The initial blood tests showed mild thrombocytosis (platelets: 538 × 10^9^/L), but otherwise unremarkable, including normal hemoglobin. The remaining diagnostic investigation showed normal chest X-ray and normal bilateral mammography and upper digestive endoscopy with chronic gastritis with positive *Helicobacter pylori *(HP) infection and colonoscopy with diverticulosis and mild hemorrhoidal disease. She was treated with antibiotic therapy for HP infection. She reports later new-onset symptoms of diffuse abdominal pain and diarrhea (without mucus or blood), associated with nausea, profuse nocturnal sweating, and progressive worsening of previous symptoms, which is why she resorted to the emergency department.

On physical examination, she was conscious and well-oriented, with mucocutaneous pallor, hydrated, normotensive and normocardic, and febrile (tympanic temperature: 38.1°C). Her abdomen was slightly painful on deep palpation, especially in the lower quadrants. The initial diagnostic workup with complete blood count showed microcytic hypochromic anemia (hemoglobin: 9 g/dL), mild leukocytosis (10.74 × 10^9^/L) with a predominance of neutrophils (85.1%), and worsening of previous thrombocytosis (platelets: 704 × 10^9^/L). The remaining laboratory tests (Table 1) showed mild hyponatremia, elevated C-reactive protein (CRP), low iron with very high ferritin values, and elevated alanine transaminase (ALT), aspartate transaminase (AST), alkaline phosphatase (ALP), and gamma-glutamyl transferase (GGT) with normal total bilirubin, prothrombin time (PT), and international normalized ratio (INR).

**Table 1:** Complementary laboratory results CRP: C-reactive protein, TIBC: total iron-binding capacity, ALT: alanine transaminase, AST: aspartate transaminase, ALP: alkaline phosphatase, GGT: gamma-glutamyl transferase, LDH: lactate dehydrogenase, TSH: thyroid-stimulating hormone

Laboratory test	Results	Reference range
Urea	26 mg/dL	17-43 mg/dL
Creatinine	0.67 mg/dL	0.6-1 mg/dL
Sodium	132 mmoL/L	135-145 mmoL/L
Potassium	4.1 mmol/L	3.5-5.1 mmol/L
CRP	189.8 mg/L	<10 mg/L
Iron	15 ug/mL	70-180 ug/mL
Ferritin	1,072.4 ng/mL	4.63-204 ng/mL
TIBC	156 ug/dL	250-425 ug/dL
Acid folic	5.9 ng/mL	3.1-20.5 ng/mL
Vitamin B12	858 pg/mL	187-883 pg/mL
ALT	62 UI/L	7-45 UI/L
AST	51 UI/L	8-35 UI/L
ALP	521 UI/L	30-120 UI/L
GGT	207 UI/L	<38 UI/L
Total bilirubin	0.43 mg/dL	0.3-1.2 mg/dL
LDH	189 UI/L	125-220 UI/L
TSH	1.17 UI/mL	0.35-4.94 UI/mL

Tests for *Clostridioides difficile *infection were negative. Viral serology such as hepatitis B, hepatitis C, and HIV, as well as autoimmunity markers, was also negative.

**Figure 1:**
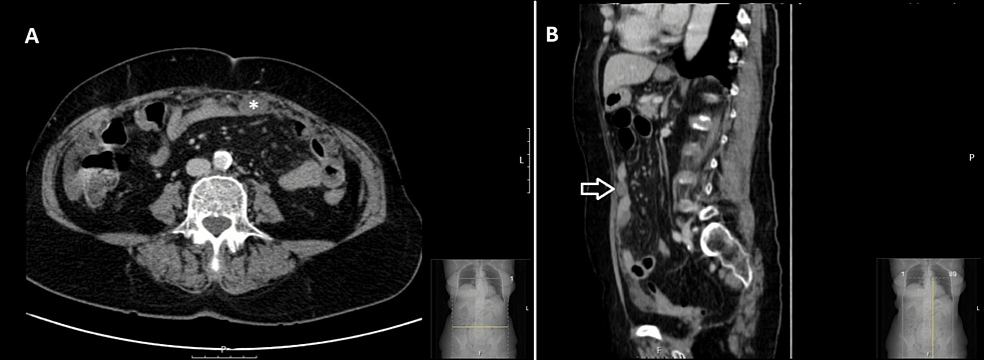
Axial (A) and sagittal (B) CT scan showing a peritoneal implant measuring 23 × 14 mm (asterisk in A and arrow in B), located in the periumbilical plane, slightly lateralized to the left CT: computed tomography

An imaging study with abdominal and pelvic computed tomography (CT) scan revealed marked tissue thickening of an irregular and striated configuration of the leaflets and peritoneal reflections, in an omental cake pattern, associated with a small amount of heterogeneous fluid in the peritoneal cavity, findings highly suspicious of peritoneal carcinomatosis. The largest peritoneal implant measuring 23 × 14 mm is located in the periumbilical plane, slightly lateralized to the left (Figure 1).

**Figure 2:**
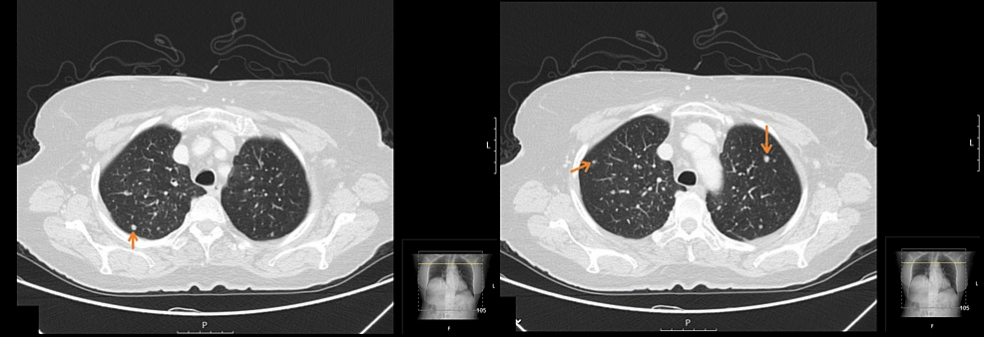
Axial CT scan of the thorax showing multiple bilateral pulmonary nodules (arrows) with less than a centimeter in size CT: computed tomography

Chest CT scan showed multiple bilateral pulmonary nodules with millimetric dimensions, without fully specific characteristics (Figure 2).

The remaining diagnostic workup highlighted tumor markers with elevated cancer antigen 125 (CA-125) with a serum value of 4,239.6 U/mL (normal range falls between 0 and 35 U/mL). A full-body positron emission tomography (PET) scan also showed thickening of the peritoneal leaflets and several solid pulmonary nodules, bilaterally distributed, with no appreciable metabolic translation. For diagnosis, a CT-guided core needle biopsy of a peritoneal implant was performed. The immunohistochemical study of the cult-guided biopsy showed fragments of mesothelial proliferation compatible with malignant peritoneal mesothelioma.

The patient showed a clinical decline and did not receive any systemic treatment, surgery, or radiotherapy and was transitioned to comfort care. Unfortunately, the patient died about a month later.

## Discussion

MPM can present as a localized abdominal disease; however, most cases have diffuse peritoneal involvement. It can also extend into the pleural cavity, causing a pleural effusion. Metastasis to abdominal and pelvic lymph nodes rarely occurs, and extra-abdominal metastases are very uncommon [6]. The diffuse disease is highly aggressive and associated with high morbidity and mortality.

Some cases of paraneoplastic phenomena have been described in the literature, including fever, thrombocytosis, and malignancy-related thrombosis [7-9]. A recent study also shows that the presence of baseline thrombocytosis has been shown to be an independent factor strongly associated with reduced survival in patients with diffuse MPM [1].

The authors described a clinical case with an aggressive presentation of MPM. This case presents with constitutional symptoms with months of evolution and only later abdominal manifestations. This contributed to a delay in diagnosis, culminating in a very advanced disease, which led to a poor prognosis for the patient. The existence of baseline thrombocytosis should be noted; however, once again, it is a nonspecific parameter and is associated with multiple other pathologies. In this case, there was also evidence of some paraneoplastic phenomena (fever and thrombocytosis) and extra-abdominal disease, which, according to the literature review, appears to be very rare [10].

The existence of radiological changes (usually CT scan) demonstrating a diffuse abdominal process compatible with malignancy should lead to differential diagnoses such as ovarian carcinoma or peritoneal carcinomatosis. There are no valuable tumor markers in the diagnosis of MPM. CA-125 is often elevated; however, this marker alone is not specific and is best used to monitor disease recurrence or progression [11]. Although the diagnosis of mesothelioma can be made cytologically, in general, cytological analysis of ascitic fluid has limited diagnostic utility. As in the case presented, CT-guided core needle biopsy or laparoscopic biopsy may both provide sufficient material to make a definite diagnosis. Features seen on hematoxylin and eosin-stained sections and immunohistochemical staining characteristics usually allow the differentiation of mesothelioma from other tumors.

Regarding treatment, there is still no consensus on the best therapeutic approach for MPM. In select patients, the use of cytoreductive surgery and hyperthermic intraperitoneal chemotherapy (CRS-HIPEC) is associated with better outcomes and survival. Patients with inoperable MPM or a high risk of early recurrence can be offered several systemic treatments, including chemotherapy, with new approaches emerging [12]. Without treatment, the median overall survival is six months [12].

Unfortunately, in the reported case, there were no treatment options, and the patient died a few months after the onset of symptoms.

## Conclusions

In this case report, the authors highlight the diagnosis of malignant peritoneal mesothelioma in a female patient without a history of asbestos exposure, which presented with nonspecific symptoms, the presence of extra-abdominal disease, and thrombocytosis as a paraneoplastic phenomenon.

Owing to the rarity of this disease and its ambiguous, nonspecific signs or symptoms, malignant peritoneal mesothelioma is difficult to diagnose, and the challenging diagnostic path worsens the prognosis. At the time of diagnosis, the majority of patients with MPM will have advanced disease, which seriously affects the prognosis of these patients.

Therefore, early diagnosis of this disease remains an important issue. Survival of patients with MPM may improve with targeted treatments and an early diagnosis.
